# Sex and Ceruloplasmin Modulate the Response to Copper Exposure in Healthy Individuals

**DOI:** 10.1289/ehp.7134

**Published:** 2004-08-17

**Authors:** Marco A. Méndez, Magdalena Araya, Manuel Olivares, Fernando Pizarro, Mauricio González

**Affiliations:** Institute of Nutrition and Food Technology, University of Chile, Santiago, Chile

**Keywords:** copper exposure, discriminant analysis, healthy individuals, principal component analysis

## Abstract

Previous studies indicated that sex might influence the response to copper exposure. Ceruloplasmin (Cp) is an indicator of Cu status, but it is not clear whether and how it reflects changes of Cu status among healthy individuals. In this study, 82 apparently healthy women and men were chosen from 800 individuals because their Cp values belonged to the higher and lower 10% of the group Cp distribution curve. Before and after receiving a supplement of 10 mg Cu/day (upper limit of daily intake) for 2 months, we performed blood and urinary biochemical measurement of potential Cu markers. We used principal component analysis and linear discriminant analysis to identify blood and/or urinary Cu indicators that showed a differential response to copper. Results showed that Cp values in serum represent a reliable indicator to differentiate subgroups within the normal population in their response to Cu exposure. The response depends on Cp values and on sex, such that women with higher and men with lower Cp values exhibit the greatest response.

Copper is required for the function of several cuproenzymes; therefore, its presence is essential for different physiological functions (Linder 1991). Cu, however, is able to generate free radicals and oxidize cellular components through its redox activity (Aust et al. 1985). These conflicting properties demand a close regulation of the metal at the organism level. Effects associated with severe lack or excess Cu are well described in genetic conditions, such as Menkes disease (Chelly et al. 1993; Mercer et al. 1993; Tanzi et al. 1993; Vulpe et al. 1993) and Wilson’s disease (Bull et al. 1993; Tanzi et al. 1996). In contrast, much less is known about relevant biological effects associated with situations when no excess or deficit of Cu is present (Davis 2003; Hambidge 2003). It is well known that serum ceruloplasmin (Cp) and Cu values are higher in young children and increase during even mild inflammatory/infectious processes, but their relationships to Cu intake and markers of Cu status are not clear, and available data suggest that they modify only when exposure changes by several orders of magnitude (Araya et al. 2003b, 2003c). It is still not clear whether marginal or moderate changes in Cu exposure may result in adverse effects to human health because there are no sensitive indicators of marginal changes in Cu status and because early functionally relevant responses are not well defined.

With the aim of improving our understanding about the early effects of Cu on human health, we have conducted a series of studies on asymptomatic adults undergoing controlled Cu exposure. This varied between approximately 3 and 10 times the customary dietary intake. Clinical trials showed that nausea is the earliest and most frequent response, and we used the generated data to calculate the dose–response curve to acute Cu exposure (Araya et al. 2003a, 2003b, 2003c; Olivares et al. 2001; Pizarro et al. 2001). A community survey in which participants ingested between 0.9 and 10 mg Cu/day for 2 months [the concentration defined as tolerable daily intake (TDI) of Cu ingestion for humans; Araya et al. (2003c)] allowed us to describe the full range of responses to Cu exposure, showing that there are more gastrointestinal responses (mainly nausea) with increasing Cu exposure (Cu concentration in water) and that these responses diminish with time, suggesting adaptation (Araya et al. 2004). In all of these studies, the statistical analyses suggested that the variable sex influenced the results.

No changes in biochemical blood parameters were detected in a previous study in which healthy participants were exposed to up to 6 mg Cu/L water, which represented as much as 14 mg Cu/day on occasional days depending on the volume of fluids ingested (Araya et al. 2003c). To further assess the homeostatic responses to Cu exposure, in a recent study we assessed the effects of exposing asymptomatic adults grouped by their Cp values to the TDI for 2 months, administered as a single daily supplement. A series of biochemical responses of blood and urinary potential Cu indicators were measured, and their detailed analysis will be reported elsewhere (Araya et al. unpublished data). The existing evidence is insufficient to determine the appropriateness of the different indicators proposed to assess Cu status among apparently normal individuals; in this article we present a multivariate strategy to evaluate a series of proposed indicators for their capacity to identify differences within the apparently healthy group. We performed our analysis as a function of sex, including the relative importance of each measurement before and after a Cu supplementation period.

## Materials and Methods

The study was a prospective controlled trial in healthy adults. Participants were 18–50 years of age, and approximately 50% were younger and older than 30 years of age. One-half of them were women who were not pregnant and did not get pregnant throughout the study. The need for volunteers was advertised in the southeastern area of Santiago; potential participants received detailed information about the protocol, and those who agreed to participate signed an informed consent before we formed the study groups. The Committee on Ethics for Human Research, Institute of Nutrition and Food Technology, University of Chile, approved the protocol. All individuals received 10 mg Cu/day administered under direct supervision as two gelatin-coated 5-mg Cu tablets (as Cu sulfate). This dose was chosen based on two criteria. First, Pratt et al. (1985) showed no significant changes in hematocrit, triglycerides, serum glutamic oxaloacetic transaminase (GOT), gamma glutamyltransferase (GGT), lactate dehydrogenase, cholesterol, or alkaline phosphatase in adult humans after administering 10 mg Cu/day as Cu gluconate or placebo capsules for 12 weeks. Second, the upper limit (10 mg Cu/day) is defined as the maximum intake from food, water, and supplements that is unlikely to pose risk of adverse health effects from excess Cu in almost all (97.5%) apparently healthy individuals, in an age- and sex-specific population group (Institute of Medicine 2001). We hypothesized that individuals may have a differential response to Cu supplementation depending on their position in the serum Cp distribution curve, considering a lower value as an index of long-term low intake. Dietary surveys assessing total daily Cu intake from food and water in Chile have revealed that 16.4% of men and 33.3% of women between 20 and 60 years of age are below the estimated average requirements (Olivares et al. 2004). Accordingly, 800 apparently healthy individuals were screened for their serum Cp protein, and 82 individuals that represented the 10% higher (high-Cp group) and 10% lower (low-Cp group) values in the Cp distribution curve were assessed (*n* = 41 for each group). Inclusion criteria were *a*) being free of acute infectious/inflammatory processes (C-reactive protein < 0.8, as indicated by the kit manufacturer; *b*) white cell count in a hemogram < 12,000 cells/mL, the lower limit of the normal range (Dallman 1977) for chronic illnesses and for chronic multimedication that may interfere with the study.

Before and after the 2-month Cu supplementation, we performed blood and urine studies. These studies included 18 potential markers of Cu status chosen from published data and our experience. Studies in blood included measurement of Cu in serum by atomic absorption spectrometry (model 2280; Perkin Elmer, Norwalk, CT, USA) and in peripheral mononuclear cells (MNCs; model SIMAA 6100; Perkin Elmer); Cp protein measured by nephelometry (array protein system; Beckman Instruments Inc., Brea, CA, USA); liver enzymes [serum GOT, serum glutamic-pyruvic transaminase (GPT), and GGT] determined using a commercial kit (Boehringer Mannheim, Mannheim, Germany); homocysteine values determined using an IMX system homocysteine kit (Abbott Laboratories, Diagnostic Division, Abbott Park, IL, USA); zinc–Cu superoxide dismutase (SOD) activity in erythrocytes measured by a Bioxytech SOD-525 Assay (OXIS International Inc, Portland, OR, USA); and glutathion measured in peripheral MNCs using a glutathione assay kit (Calbiochem; Cayman Chemical Company, Ann Arbor, MI, USA). Studies in urine included measurement of urinary Cu excretion after a chelator challenge with 300 mg 2,3-dimercaptopropane-1-sulfonate (DMPS or Dimaval; Heyl Laboratory, Berlin, Germany), within 3 days before and after supplementation beginning and end. Calculating sample size using α at 5% and power at 80%, we needed 35–45 individuals per group to detect a delta of 0.5 standard deviations in the biochemical measurements that were planned.

Data were analyzed using SYSTAT 5.0 (SYSTAT, Inc., Evanston, IL, USA; Wilkinson 1996). All data were log transformed in order to meet the assumptions of normality of data. Because study groups were formed on the basis of individuals’ serum Cp values (high-Cp and low-Cp), data were first assessed by univariate analysis at the beginning and end of the supplementation period to determine whether groups formed by individuals’ Cp values were significantly different. The integral (multivariate) response to Cu exposure (defined as the response of all biochemical measurements at the same time) was explored by multivariate analysis using principal component analysis (PCA) and linear discriminant analysis (LDA). PCA is a multivariate statistical tool that simplifies complex data sets by transforming the original variables into new independent and uncorrelated variables named principal components, which explain the observed variability in decreasing order. Thus, the first components concentrate maximal information (variance explained) about the analysis; additionally, for each component there is an eigenvalue with an associated variance value (explained variance). On the other hand, we used LDA as a classification function to calculate scores for each variable in the different groups; LDA permits evaluating whether there are significant relationships between qualitative variables or classes (in this case, sex and low-Cp and high-Cp groups) and quantitative predictor variables (in our case, eigenvalues of each biochemical variable). Because we knew the classes, we built a linear discriminant function to estimate the goodness of this classification within each class. A matrix originated by PCA served as the basis for LDA input data; in all analyses, we added eigenfactors until obtaining close to 80% of variability in the model. Thus, the LDA output allowed assessing *a*) whether sex and Cp levels (low-Cp and high-Cp) differences are associated with responses to copper; and *b*) the integral (multivariate) response of individuals to Cu exposure. Additionally, because the discriminant function was applied to the same sample used to derive it, we used both cross-validation and jackknife procedures to obtain unbiased estimates (Hair et al. 1992). Using these procedures, we obtained a classification matrix that allowed evaluating the performance of the defined classes (sex and low-Cp and high-Cp groups), verifying which individuals had high values of correct classifications. As a control, we also assessed whether the analyses performed using both cross-validation and jackknife procedures yielded the same values of correct classifications; because no differences were found, we only show the results of the jackknife matrix. All the LDA data showed normal multivariate distribution, which was evaluated using the Sen and Puri test (Sen and Puri 1968).

## Results

### Univariate analysis.

**Figure 1:**
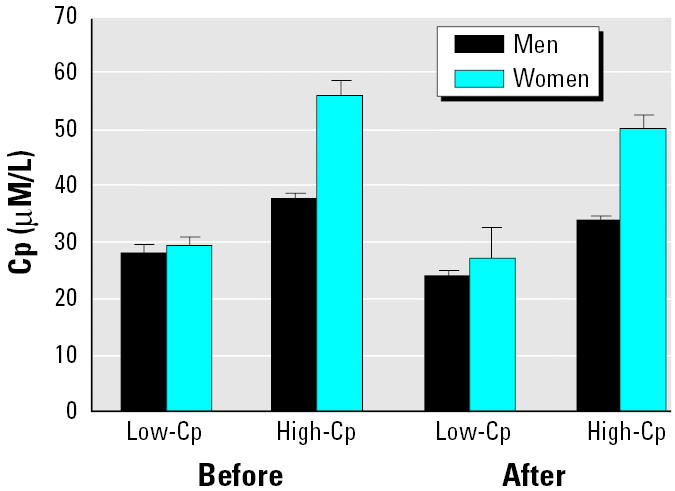
Cp concentration (media ± SE) in men and women before and after Cu supplementation grouped by Cp concentrations of individuals.

As expected, Cp values of individuals grouped by Cp group (low-Cp and high-Cp) and sex showed significant differences at the beginning [analysis of variance (ANOVA), Cp group: *F* = 103.99, df = 1, *p* < 0.0001; sex: *F* = 22.256, df = 1, *p* < 0.0001; interaction Cp group × sex: *F* = 10.591, df = 80, *p* < 0.002; Figure 1], and end of treatment (ANOVA, Cp group: *F* = 126.710, df = 1, *p* < 0.0001; sex: *F* = 22.256, df = 1, *p* < 0.0001; interaction Cp group × sex: *F* = 10.591, df = 80, *p* < 0.008; Figure 1).

### Multivariate analysis.

As a first step, we explored whether differences existed when all biochemical measurements were considered at the same time (integral response), assorting individuals both by sex and Cp groups. PCA showed that the first four components explained 68.62% of the variability; in the first component, ferritin, GGT, and GPT obtained the highest loading value (Table 1), whereas serum Zn and Cu and Cu in MNCs obtained the lowest values. LDA, using the matrix obtained from the first six PCA components (which explained > 80.8% of the variability observed) and using sex as a classification variable, revealed statistically significant differences between sexes (Wilks’s lambda = 0.423; *F* = 17.043; df = 6, 75; *p* < 0.0001). A discriminant classification matrix showed high values of correct classifications (80% for men, 88% for women), indicating that there were differences related to sex associated with the parameters evaluated. In view of these results, the next analyses were performed separately on women and men, before and after Cu supplementation.

### Before Cu supplementation.

In women, PCA showed that the first four components explained 70.6% of the variability. In the first component, ferritin, GGT, and GPT obtained the highest loading value (Table 2). Among men, PCA showed that the first four components explained 70.38% of the variability. Compared with women, the relative importance of each element in the first component somewhat differed: GPT and GGT were included, but not ferritin, and DMPS (1–4 hr) was among the variables with the highest loading value (Table 2). The LDA analysis performed using high- and low-Cp group as classificatory variables revealed that these groups were statistically different (Wilks’s lambda = 0.5136; *F* = 5.3673; df = 6, 34; *p* < 0.0005), showing high values of correct classifications in the classification matrix (low-Cp group, 80%; high-Cp group, 81%). This analysis also shows that although differences between the Cp groups were significant (Wilks’s lambda = 0.660; *F* = 2.919; df = 6, 34; *p* < 0.020), the values of correct classifications were lower in men than in women (low-Cp group, 70%; high-Cp group, 67%).

### After Cu supplementation.

In women, PCA showed that the first four components explained 73.27% of the variability. In the first component, as before, Cu supplementation, GGT, ferritin, and GPT obtained the highest loading value (Table 2). The LDA showed significant differences between the Cp groups (Wilks’s lambda = 0.574; *F* = 4.074; df = 6, 33; *p* < 0.0003) and a classification matrix with values of correct classifications > 50% (low-Cp group, 76%; high-Cp group, 68%). Among men, PCA showed that the first components explained 65.91% of the variability. In the first component, GGT, GPT, and GOT showed the highest loading value, showing that in comparison with analysis before Cu supplementation, GOT replaced DMPS (1–4 hr; Table 2). LDA in men also showed differences between Cp groups (Wilks’s lambda = 0.584; *F* = 2.496; df = 8, 32; *p* < 0.016), and as in the case of women, values of correct classifications were > 50% (low-Cp group, 60%; high-Cp group, 67%). Both in women and in men, the percentages of correct classification were lower in comparison with the figures obtained before Cu supplementation.

## Discussion

It is well known that serum Cp and Cu vary responding to rather minor inflammatory and infectious events; at the same time, these indicators are used to assess changes in Cu status in pathological situations. To what extent they may reflect mild yet relevant changes of Cu status among apparently healthy individuals is still a matter of debate (Araya et al. 2003b, 2003c; Davis 2003; Hambidge 2003; Kehoe et al. 2000). In this study, participants were healthy and remained clinically healthy during the 2-month controlled Cu exposure. Serum activities of aminotranferases are the traditional biochemical blood measurements used clinically to assess liver function. Participants received a daily Cu dose defined as the upper safe limit for human consumption, so toxic responses were not expected; liver aminotranferases were evaluated to satisfy ethical considerations. We detected no responses that may represent toxic effects of the Cu dose used.

Both the univariate and bivariate analyses support the idea that Cp values in serum represented a reliable indicator of Cu status responding to chronic Cu exposure and that sex was indeed a factor that modulated the response. It is interesting that both in women and in men and both before and after Cu supplementation, GGT and GPT were always included in the first component with high loading values; ferritin was included only among women, whereas urinary Cu excretion after DMPS challenge was included among men. Because it is difficult to interpret these differences with the present data and there is little experience with DMPS challenge among normal populations, these findings deserve further research. The LDA also showed, both in women and in men, that the biochemical indicators measured were significantly associated with the Cp values; the classification matrix showed that the correct assignment values were higher among women (~ 80%), whereas among men they reached values of about 70%, suggesting that the Cp value is a good indicator to separate the groups, and a reliable descriptor of the integral response to Cu exposure, but it may be more sensitive for women that for men.

By the end of the Cu exposure period, in both sexes the first four PCA components explained a lower proportion of variance (73.3% in women and 65.9% in men), suggesting that variability increased in the groups after Cu supplementation. It is intriguing that, among women, ferritin decreased its loading value whereas GGT became the most relevant variable (Table 2). Among men, GGT is also the main variable explaining the variance, and the three transaminases as a whole (GGT, GPT, and GOT) are the factors with the highest loading values. This is a relevant finding because these enzymes classically change in hepatic diseases, but it is not clear that they respond to subclinical situations (Cashman et al. 2001; Jones et al. 1997; Lowe et al. 2002; Nayak et al. 2003; Olusi et al. 2003); even accepting that they may respond to other illnesses, our results support their use to monitor potential adverse effects of Cu in the liver.

## Conclusion

We conclude that Cp values in serum represent a reliable indicator of Cu status and of the host response to Cu exposure. This information is relevant to risk assessment studies of Cu effects in human health and environmental epidemiology. This response depends on sex and also on the Cp value, such that women with higher Cp values and men with lower Cp values exhibit the greatest response. Why women respond differently than men and why apparently healthy individuals respond differently depending on their Cp values is not clear; ongoing studies are currently exploring these aspects.

## Figures and Tables

**Table 1:** Values (loadings) in the first four PCAs for each of the 18 variables assessed in adult individuals before Cu supplementation.

Variables	PC1	PC2	PC3	PC4
Hemoglobin	0.104	0.049	−0.184	−0.018
Ferritin	1.347	0.077	−2.606	1.396
Homocysteine	0.127	−0.104	−0.177	−0.719
Serum Cu	−0.003	0.418	0.917	−0.300
Serum Fe	0.169	0.578	−0.365	−0.397
Serum Zn	−0.003	0.002	−0.086	0.247
Cu in MNC	−0.005	−0.066	0.130	0.075
Fe in MNC	−0.013	−0.143	0.128	−0.294
Zn in MNC	0.017	−0.099	−0.023	−0.105
GOT	0.542	−0.437	1.328	0.929
GPT	0.945	−1.187	1.111	1.372
GGT	1.680	0.659	1.342	−1.571
SOD	0.223	0.264	0.395	1.491
DMPS	0.261	2.124	−0.240	0.025
DMPS 0–4 hr	−0.333	0.502	0.782	2.621
DMPS 5–24 hr	−0.052	0.830	0.619	−0.217
DMPS 0–24 hr	−0.318	1.199	0.443	1.185
Urinary Cu	0.058	0.648	0.038	0.297
Percent variance explained	29.08	18.11	12.8	9.650

Value for DMPS is Cu excreted during the indicated time period after administration of 300 mg DMPS.

**Table 2:** Loadings from the first PCAs studied before and after Cu supplementation.

	Before		After	
Variables	Women	Men	Women	Men
Hemoglobin	0.059	0.006	0.021	−0.029
Ferritin	1.161*^a^*	−0.346	1.553*^a^*	0.428
Homocysteine	−0.034	−0.147	0.147	0.293
Serum Cu	0.263	0.038	0.295	0.132
Serum Fe	0.132	0.093	0.130	−0.106
Serum Zn	0.006	0.058	−0.033	−0.125
Cu in MNC	0.001	−0.074	0.116	0.090
Fe in MNC	−0.005	−0.112	0.076	0.102
Zn in MNC	0.018	−0.094	0.072	0.143
GOT	0.679	−0.993*^a^*	0.722	0.870*^a^*
GPT	0.848*^a^*	−1.640*^a^*	0.781*^a^*	0.885*^a^*
GGT	1.656*^a^*	−1.197*^a^*	2.175*^a^*	2.540*^a^*
SOD	0.326	0.012	−0.152	−0.321
DMPS	0.716	0.908	0.512	0.844
DMPS 0–4 hr	0.165	0.675		
DMPS 5–24 hr	0.104	0.206		
DMPS 0–24 hr	−0.229	0.512	0.472	−0.441
Urinary Cu	0.100	0.279	0.497	0.337
Percent variance explained	31.58	29.58	29.57	26.19

Values for DMPS are Cu excreted during the indicated time period after administration of 300 mg DMPS.

a Variables with highest loading in the first component.
